# Enrichment of Comammox and Nitrite-Oxidizing *Nitrospira* From Acidic Soils

**DOI:** 10.3389/fmicb.2020.01737

**Published:** 2020-07-22

**Authors:** Yu Takahashi, Hirotsugu Fujitani, Yuhei Hirono, Kanako Tago, Yong Wang, Masahito Hayatsu, Satoshi Tsuneda

**Affiliations:** ^1^Department of Life Science and Medical Bioscience, School of Advanced Science and Engineering, Waseda University, Tokyo, Japan; ^2^Department of Biological Sciences, Faculty of Science and Engineering, Chuo University, Tokyo, Japan; ^3^Institute of Fruit Tree and Tea Science, National Agriculture and Food Research Organization (NARO), Shimada, Japan; ^4^Institute for Agro-Environmental Sciences, National Agriculture and Food Research Organization (NARO), Tsukuba, Japan

**Keywords:** acidic soil, nitrifying bacteria, nitrification, *Nitrospira*, comammox, cultivation, enrichment, low pH

## Abstract

In agricultural soils fertilized with a high amount of ammonium nitrogen, the pH decreases because of the oxidation of ammonia by nitrifiers. Molecular-based analyses have revealed that members of the genus *Nitrospira* dominate over other nitrifiers in some acidic soils. However, terrestrial *Nitrospira* are rarely cultivated and little is known about their ecophysiology. In addition, recent studies discovered a single microbe with the potential to oxidize both ammonia and nitrite (complete ammonia oxidizer; comammox) within *Nitrospira*, which had been previously recognized as a nitrite oxidizer. Despite their broad distribution, there are no enrichment samples of comammox from terrestrial or acidic environments. Here, we report the selective enrichment of both comammox and nitrite-oxidizing *Nitrospira* from the acidic soil of a heavily fertilized tea field. Long-term enrichment was performed with two individual continuous-feeding bioreactors capable of controlling ammonia or nitrite concentration and pH. We found that excessive ammonium supply was a key factor to enhance the growth of comammox *Nitrospira* under acidic conditions. Additionally, a low concentration of nitrite was fed to prevent the accumulation of free nitrous acid and inhibition of cell growth under low pH, resulting in the selective enrichment of nitrite-oxidizing *Nitrospira*. Based on 16S rRNA gene analysis, *Nitrospira* accounting for only 1.2% in an initial soil increased to approximately 80% of the total microorganisms in both ammonia- and nitrite-fed bioreactors. Furthermore, *amoA* amplicon sequencing revealed that two phylotypes belonging to comammox clade A were enriched in an ammonia-fed bioreactor. One group was closely related to previously cultivated strains, and the other was classified into a different cluster consisting of only uncultivated representatives. These two groups coexisted in the bioreactor controlled at pH 6.0, but the latter became dominant after the pH decreased to 5.5. Additionally, a physiological experiment revealed that the enrichment sample oxidizes ammonia at pH <4, which is in accordance with the strongly acidic tea field soil; this value is lower than the active pH range of isolated acid-adapted nitrifiers. In conclusion, we successfully enriched multiple phylotypes of comammox and nitrite-oxidizing *Nitrospira* and revealed that the pH and concentrations of protonated N-compounds were potential niche determinants.

## Introduction

Nitrification is an important reaction composing the global nitrogen cycle. In soil ecosystems, nitrification acts as a production route of nitrate, a nitrogen source of soil plants. Despite the importance of nitrification, supplementation with excessive nitrogen fertilizers over-activates nitrification and induces environmental problems. Too much nitrate produced by nitrification leaches into groundwater, causing pollution and the economic loss of fertilizers (Schlesinger, 2009). Furthermore, excessive nitrification also causes the release of nitrous oxide, a greenhouse gas (Wrage et al., 2001) and soil acidification (Vitousek et al., 1997). In particular, acidic soils (defined as pH <5.5) are known to show the same or higher nitrification rate as neutral soils (Booth et al., 2005). Moreover, soils with lower pH were reported to generate more nitrous oxide (Mørkved et al., 2007; Liu et al., 2010; Bakken et al., 2012).

Traditionally, nitrification had been thought to consist of two independent steps: ammonia oxidation (NH_3_ to NO_2_^–^) and nitrite oxidation (NO_2_^–^ to NO_3_^–^). The first step is catalyzed by ammonia-oxidizing bacteria (AOB) and ammonia-oxidizing archaea (AOA), while the second step is carried out by nitrite-oxidizing bacteria (NOB). Molecular approaches revealed that AOA predominated among ammonia oxidizers in soils (Leininger et al., 2006) including acidic soils (Nicol et al., 2008; Zhang et al., 2012). In addition, a strain of acidophilic AOA, “*Candidatus* Nitrosotalea devanaterra” was isolated from acidic soil (Lehtovirta-Morley et al., 2011) and physiologically characterized (Lehtovirta-Morley et al., 2014). Although these studies supported the importance of AOA in nitrification in acidic soils, AOB was reported to outnumber AOA in some acidic soils (Long et al., 2012; Petersen et al., 2012; Wertz et al., 2012). Furthermore, a strain of acid-tolerant AOB, “*Candidatus* Nitrosoglobus terrae” was isolated from the acidic soil of a tea field (Hayatsu et al., 2017). This study proved that the phylogenetically novel AOB classified into *Gammaproteobacteria* were more abundant than other ammonia oxidizers in some tea fields (Hayatsu et al., 2017). In contrast, for acidophilic NOB, *Nitrobacter* sp. Io acid was isolated from acidic forest soil (Hankinson and Schmidt, 1988). All of the acid-tolerant isolates of NOB were classified into the genus *Nitrobacter*: *Nitrobacter* sp. Io acid (Hankinson and Schmidt, 1988), *Nitrobacter winogradskyi* (Bock and Heinrich, 1969), and *Nitrobacter* strain NHB1, co-cultured with AOB (De Boer et al., 1991). However, *Nitrospira*, a different genus of NOB, outnumbers *Nitrobacter* in other acidic soils (Wertz et al., 2012; Stempfhuber et al., 2017). Moreover, in several studies cultivating samples from wastewater treatment plants and soilless medium-based horticulture systems, *Nitrospira* became dominant in acidic cultures at pH <5 (Tarre and Green, 2004; Cytryn et al., 2012). Although these cultures were not from soils, such previous reports support that uncultivated *Nitrospira* could contribute to nitrification in acidic environments.

Recent studies found a complete ammonia oxidizer (comammox) in the genus *Nitrospira*; this novel bacterium oxidizes both ammonia and nitrite in a single cell (Daims et al., 2015; van Kessel et al., 2015). Comammox *Nitrospira* is phylogenetically diverse and separated into two sister clades named as clade A and B, based on ammonia monooxygenase subunit A (*amoA*) gene sequence (Daims et al., 2015). Since this discovery, many studies have investigated the potential metabolisms and environmental distributions of comammox based on molecular biological techniques such as metagenomics (Palomo et al., 2016, 2018; Bartelme et al., 2017; Camejo et al., 2017; Wang et al., 2017; Orellana et al., 2018). Based on these metagenomic datasets, quantitative PCR primers targeting comammox were designed (Pjevac et al., 2017). In a study using this primer set, comammox was reported to be the most dominant in nitrifying bacteria communities in acidic soils at pH 4.0–7.0 (Hu and He, 2017). While the importance of comammox and nitrite-oxidizing *Nitrospira* in acidic soils has been clarified as described above, cultivation of these bacteria has hardly been attempted. Only one study obtained an isolate of comammox, *Nitrospira inopinata*, from a biofilm sustained in thermal waters at pH 7.5 (Daims et al., 2015; Dimitri et al., 2017). Also, “*Candidatus* Nitrospira nitrosa” and “*Candidatus* Nitrospira nitrificans” were enriched from a recirculation aquaculture system biofilter in a sequencing batch reactor operated at pH 6.99 (van Kessel et al., 2015). Other culture samples of comammox were obtained from wastewater treatment plants (Camejo et al., 2017; Roots et al., 2019) nitrifying granules (Fujitani et al., 2013), and river sediments (Yu et al., 2018). On the basis of these cultivation researches and genomic studies, physiological and biochemical characteristics of comammox *Nitrospira* have been speculated. Comammox *Nitrospira* is presumed to have advantage over other nitrifiers under low dissolved oxygen concentration and adapted to slow growth in oligotrophic environments (Koch et al., 2019). However, all the comammox cultures were incubated with a neutral or alkaline medium. For this reason, culture samples allowing the study of the acid-adaptation and ecology of comammox *Nitrospira* in acidic soils have not been obtained yet.

In this study, we focused on the cultivation of comammox and nitrite-oxidizing *Nitrospira* from acidic soils. The soil sample was collected from a tea field. Tea fields are supplemented with higher amount of N fertilizer than typical croplands and the excessive fertilization causes N-related problems, such as nitrate leaching (Hirono et al., 2009), soil acidification, and N_2_O production (Tokuda and Hayatsu, 2004). Using two types of bioreactors, we enriched both comammox and nitrite-oxidizing *Nitrospira* at pH 5.5. During a long-term enrichment process over 2 years, the microbial community was analyzed by 16S rRNA gene and *amoA* gene amplicon sequencing. Furthermore, ammonia oxidation activity tests using the enrichment samples were performed to investigate adaptation to different pH and ammonia concentrations.

## Materials and Methods

### Soil Samples

Following our previous study (Hayatsu et al., 2017) soil samples were collected in August 2017 from tea field plots supplemented with N fertilizer of 506 kg N ha^–1^ year^–1^ at Kanaya Tea Research Station, Institute of Fruit Tree and Tea Science, NARO, in Japan (34°48′28.2′′N 138°07′55.9′′E). Soil samples were immediately stored at −80°C or 4°C. Molecular analyses were performed on the samples stored at –80°C. The characteristics of the soils are shown in Supplementary Table S1. Samples stored at 4°C were used for incubation within a month of sampling.

### Continuous-Feeding Incubation

Following our previous research (Fujitani et al., 2013) two types of continuous-feeding bioreactors were set up to selectively enrich comammox and nitrite-oxidizing *Nitrospira*. A 13 g soil sample was suspended in an inorganic medium and incubated in batch culture. As biomass carriers for soil bacteria, non-woven fabrics were sunk in the medium. To attach the biomass to the non-woven fabrics, the suspended soil sample was statically incubated without exchanging the medium. The pH in the medium was manually kept under 6.0 and the concentration of NH_4_Cl in the medium was kept under 0.4 mM. After pre-incubation for a month, the non-woven fabrics and supernatant in the batch culture were transferred to a bioreactor. Subsequently, inorganic medium containing NH_4_Cl was continuously supplied into the continuous-feeding bioreactor (day 0 of continuous-feeding incubation). The biomass was maintained on the non-woven fabrics to prevent microorganisms to be washed out from the bioreactor. On day 25, part of the biomass in the NH_4_Cl-fed bioreactor was transplanted to the other bioreactor, and a medium containing NaNO_2_ was supplied continuously.

The components of inorganic medium supplied into the NH_4_Cl-fed and NaNO_2_-fed bioreactors are shown in Supplementary Tables S2, S3, respectively. The capacity of both bioreactors was 1.0 L and the inorganic media were supplied at a rate of 3.0 L day^–1^. The bioreactors were operated in a dark room maintained at 23°C and were supplied with excess oxygen by aeration. The inorganic media and pH adjustment chemicals were supplied by tubing pumps (ATTO Co., Tokyo, Japan) into the bioreactors. The influx of pH adjustment chemicals was automatically operated by digital pH controllers (Nissin Rika Co., Tokyo, Japan), which are able to measure the pH values in the bioreactors and control the flux in real time. NaHCO_3_ and HCl were used as pH adjustment chemicals for the NH_4_Cl- and NaNO_2_-fed bioreactors, respectively.

During continuous-feeding incubation of the NH_4_Cl-fed bioreactor, the NH_4_Cl concentration in the supplied medium increased in a stepwise way from 0.07 to 0.14, 0.71, 1.4, 2.1, 7.1, 18, 29, 39, and 50 mM (day 0–206). The inflow NH_4_Cl concentration was determined, following our previous study (Hayatsu et al., 2017). From day 206 to the end of the experiment, the concentration was maintained at 50 mM. The pH in the NH_4_Cl-fed bioreactor was controlled at pH 6.0 ± 0.2 from day 0 to 373. On day 374, after the stable nitrification was observed at pH 6.0, the pH value was decreased to 5.5 ± 0.2, and kept a constant level until the end of the experiment. Likewise, the concentration of NaNO_2_ in the medium supplied into the NaNO_2_-fed bioreactor was increased in a stepwise manner from 0.07 to 0.14, 0.29, 0.43, 0.71, 1.8, 2.9, and 3.9 mM (day 25–150), following our previous research (Fujitani et al., 2013). Thereafter, the concentration was maintained for a while, but was again reduced from 3.9 to 1.8, 0.71, and 0.36 mM at last from day 310 to 387 to reduce the stress on the biomass. The pH in the NaNO_2_-fed bioreactor was controlled at pH 6.0 ± 0.2 from day 0 to 303. The pH value was decreased to 5.5 ± 0.2 on day 304 and kept at a constant level until the end of the experiment.

### Chemical Analyses During Continuous-Feeding Incubation

For each culture condition, the culture solution in each bioreactor was collected and sterilized by passing through 0.22 μm polyethersulfone membrane filters (Millipore, Eschborn, Germany). These solution samples were stored at –20°C until chemical analyses to measure the total ammonia–nitrogen (NH_4_^+^ + NH_3_), nitrite–nitrogen (NO_2_^–^–N), and nitrate–nitrogen (NO_3_^–^–N) concentrations. The total ammonia–nitrogen concentration was measured using the indophenol blue method (Kandeler and Gerber, 1988) with a PowerWave HT microplate spectrophotometer (BioTek Instruments Inc., Winooski, VT, United States) using the absorbance at 630 nm as an index. The nitrite–nitrogen and nitrate–nitrogen concentrations were measured using an IC-2010 ion chromatography system (Tosoh Co., Tokyo, Japan). Based on the measured values, the concentrations of free ammonia (NH_3_) and free nitrite (HNO_2_) were calculated according to the method of a previous study (Anthonisen et al., 1976).

### Microscopic Observation

The cell suspensions were collected from non-woven fabrics in bioreactors on days 84, 228, 319, and 791. The samples were fixed and stored at –20°C until observation. The fixed samples were sonicated with a Q55 homogenizer (Qsonica LLC., Newtown, CT, United States) at 20% amplitude for 30 s to disperse bacterial aggregates. The sonicated samples were dropped onto slide glasses and stained using fluorescence *in situ* hybridization (FISH) as described in a previous study (Amann et al., 1990). Oligonucleotide probes binding specifically to 16S rRNA of *Nitrospira* lineage II, *Nitrospira* lineage I, *Nitrobacter*, and betaproteobacterial AOB were labeled with hydrophilic sulfoindocyanine dye (Cy3) (Supplementary Table S4). These probes were added to the slide glasses and hybridized at 46°C for 2.5 h. Furthermore, all bacteria were stained with SYTOX Green nucleic acid stain (Life Technologies, Carlsbad, CA, United States). The stained samples were observed with an Axioskop 2 Plus fluorescence microscope (Carl Zeiss, Oberkochen, Germany).

### DNA Extraction

Genomic DNA was extracted from the initial soil samples used for cultivation and cell suspensions collected from the bioreactors. The extraction was performed using the FastDNA SPIN Kit for Soil (MP Biomedicals, Irvine, CA, United States) according to the protocol of the manufacturer. The extracted DNA was stored at –20°C until amplicon sequencing.

### Amplicon Sequencing of 16S rRNA Gene and *amoA* Gene

Amplicon sequencing targeting the variable regions V7 and V8 of the 16S rRNA gene was performed with primers with added adapter sequences (Supplementary Table S5). The extracted genomic DNA was amplified by PCR using Ex Taq (Takara Bio Inc., Shiga, Japan). The amplicon was sequenced by the Ion Torrent Personal Genome Machine sequencer (Life Technologies, Carlsbad, CA, United States) (McDonald et al., 2012). Sequence data were processed with CLC Genomics Workbench v5.5.1 (CLC bio, Aarhus, Denmark). The barcode sequences, sequences of inappropriate length (<250 bp or >350 bp), sequences with low-quality scores (limit = 0.005), and sequences containing ambiguous nucleotides (five nucleotides at most) were trimmed. The processed sequences were analyzed using QIIME (Caporaso et al., 2010) and sequences with 98% or more homology were compiled as operational taxonomic units (OTUs) using the UCLUST algorithm (Edgar, 2010). One representative sequence was extracted from each OTU, and the sequences were assigned referring to the SILVA database version 132 (Quast et al., 2013). Sequences sharing less than 80% homology with the references were defined as unclassified. To investigate the phylogeny of OTUs in more detail, sequences of OTUs classified as nitrifiers were analyzed using the Basic Local Alignment Search Tool (BLAST) server^1^ of the National Center for Biotechnology Information (NCBI).

Likewise, adapter sequences were added to a primer pair targeting the *amoA* gene of comammox (Supplementary Table S5). PCR amplification, sequencing, and trimming procedures were the same as those for the 16S rRNA gene, except for trimming and compiling. The sequences of inappropriate length (<150 bp or >250 bp) were trimmed and sequences with homology of 97% or more were summarized as OTUs.

### Phylogenetic Analyses

The sequences of 16S rRNA genes classified into each lineage of the genus *Nitrospira* were searched on the nucleotide database of NCBI^2^. Based on these sequences and representative sequences of OTUs obtained in this study, phylogenetic analysis was performed using MEGA7 software (Shindo et al., 2006). The collected sequences were aligned and a phylogenetic tree was constructed using the neighbor-joining method (Saitou and Nei, 1987) and the bipartition confidence was evaluated at 1,000 bootstraps (Felsenstein, 1985).

The whole genome sequences of cultivated comammox, metagenome-assembled genomes (MAGs) constructed from environmental samples and clone sequences of the *Nitrospira amoA* gene detected in cultured samples were searched for in the NCBI nucleotide database, and AmoA amino acid sequences were collected. In case coding sequences (CDSs) of *amoA* were not annotated in the GenBank entry on the database, the MAG sequences were downloaded and CDSs were annotated using Prokka (version 1.13) (Seemann, 2014). To obtain AmoA amino acid sequences of enriched comammox and to correct the frame-shift of those sequences, the nucleotide sequences of OTUs were translated using the FrameBot tool (Wang et al., 2013) referring to the AmoA sequence of *Nitrospira inopinata*. The phylogenetic tree based on AmoA amino acid sequences was constructed using the same method as that for the 16S rRNA genes.

### Nitrification Experiments of NH_4_Cl-Fed Enrichment

To examine the effect of NH_4_Cl concentration on the nitrification activity, a batch culture test was performed. An enrichment sample was collected from the NH_4_Cl-fed bioreactor on day 749 and washed using centrifugation (2,900 × *g*, 10 min). The fresh inorganic medium described above was added to the sample and the bacterial pellet was dispersed with a Q55 homogenizer (Qsonica LLC, Newtown, CT, United States) for 30 s at 20% amplitude. The suspended sample was transferred into test tubes containing inorganic medium adjusted to pH 5.5, containing 0, 12.5, 25, 50, 75, 100, 200, or 300 mM NH_4_Cl. The test tubes were incubated for three days in a dark room maintained at 23°C. The test was performed with three biological replicates. The supernatant of the medium in the tubes was collected and stored at –20°C until chemical analyses.

To examine the effect of pH on nitrification activity, another batch culture test was performed. The method for preparing bacterial suspension was as described above, except for the sampling was performed on day 849. The sample was transferred into test tubes with inorganic medium containing 12.5 mM NH_4_Cl, adjusted to pH 3.0, 4.0, 5.0, 6.4, 7.5, or 9.0. As buffers of the medium, 80 mM 2-morpholinoethanesulfonic acid (MES) for pH 5.0, 4-(2-hydroxyethyl)-1-piperazine-ethanesulphonic acid (HEPES) for pH 6.4, and 25 mM N-cyclohexyl-3-aminopropanesulfonic acid (CAPS) for pH 7.5 and 9.0 were mixed. The concentration of CAPS was adjusted to the condition described in a previous report (Dai et al., 2012). The media for the pH 3.0 and 4.0 tests were unbuffered. The tubes were incubated for 2 days and collected samples were stored as described above.

### Chemical Analyses for Nitrification Experiments

The nitrite concentrations of culture samples were measured using the Griess test (Griess-Romijn, 1966) using the absorbance at 560 nm as the index. As described in a previous study (Miranda et al., 2001) nitrate concentrations were determined by reducing nitrate to nitrite with vanadium chloride (III) and measured using the Griess test. The absorbance was measured using a PowerWave HT microplate spectrophotometer (BioTek Instruments Inc., Winooski, VT, United States).

## Results and Discussion

### Water Quality During Continuous-Feeding Incubation

**FIGURE 1:**
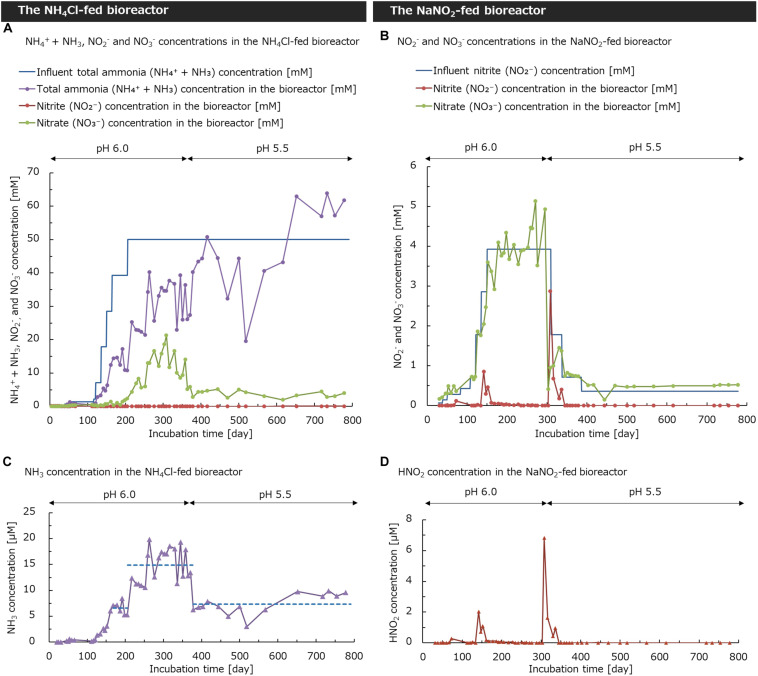
Total ammonia, nitrite, and nitrate concentrations in the **(A)** NH_4_Cl-fed bioreactor and **(B)** NaNO_2_-fed bioreactor. The theoretically calculated concentration of NH_3_ in the NH_4_Cl-fed bioreactor and HNO_2_ in the NaNO_2_-fed bioreactor are shown in **(C,D)**, respectively. The dashed lines in **(C)** show the average value of each period. The pH values in the NH_4_Cl-fed bioreactor and NaNO_2_-fed bioreactor were changed from 6.0 to 5.5 on days 374 and 304, respectively.

At the start of the continuous-feeding incubation in the NH_4_Cl-fed bioreactor, the medium was maintained at around pH 6.0. Under acidic conditions, the concentration of free ammonia (NH_3_) decreased exponentially as the pH decreased, owing to protonation from ammonia to ammonium (NH_4_^+^) (pKa = 9.25) (De Boer and Kowalchuk, 2001). Ammonia, not ammonium, is known as the substrate of ammonia monooxygenase (Suzuki et al., 1974; Galloway et al., 2008). Considering the decrease in ammonia concentration caused by protonation, an excessive amount of ammonium was supplied into the bioreactor to enrich the comammox *Nitrospira*. The concentration of NH_4_Cl in the supplied medium was increased in a stepwise way. On day 165–205, when the bioreactor was supplied with the medium containing 39 mM NH_4_Cl, the nitrate (NO_3_^–^) concentration in the bioreactor increased to 1.3 ± 0.8 mM on average, which shows an increase in nitrification activity (Figure 1A). After the further increase in supplied NH_4_Cl concentration to 50 mM, the NO_3_^–^ concentration in the bioreactor reached 11.2 ± 4.9 mM during days 206–373. Although the total ammonia (NH_4_^+^ + NH_3_) in the bioreactor was not completely oxidized to nitrate, a low concentration of NO_3_^–^ was stably produced by nitrification. Based on this result, the influent concentration of NH_4_Cl was fixed at 50 mM in the subsequent incubation. To enrich the nitrifiers adapted to a more acidic condition, we decreased the pH from 6.0 to 5.5, in which most of the isolated ammonia oxidizers cannot grow (De Boer and Kowalchuk, 2001). After the decrease in pH during days 374–791, the NO_3_^–^ concentration in the bioreactor reduced to 3.7 ± 1.0 mM, indicating the suppression of nitrification activity. However, in acidic soils, high concentration of ammonium accumulated (Supplementary Table S1). On the other hand, nitrate concentration in soils was two orders of magnitude less than that of ammonium. Thus, the culture condition with low pH and high influent concentration of ammonia could reproduce the environment in acidic soils, although ammonia in the bioreactor was not completely oxidized. Furthermore, the concentration of NO_2_^–^ was maintained at under 0.2 mM throughout the cultivation process. This result was also in accordance with the low nitrite concentration in soils (Kowalchuk and Stephen, 2001). Throughout the incubation, the measured concentration of total ammonia (NH_4_^+^ + NH_3_) in the bioreactor was not stable. This is considered to be due to the high reagent blank of the indophenol blue method (Willason and Johnson, 1986; Aminot et al., 1997; Kérouel and Aminot, 1997). Moreover, the stoichiometric ratio of ammonia consumption and nitrate generation was not consistent. This inconsistency could be caused by the consumption of ammonia as nitrogen source or denitrification converting nitrate to gaseous nitrogen.

A part of the biomass in the NH_4_Cl-fed bioreactor was transferred to the NaNO_2_-fed bioreactor on day 25. The medium in the bioreactor was maintained at around pH 6.0. Under low pH conditions, the concentration of free nitrous acid (HNO_2_) increases because of the protonation of NO_2_^–^ (pKa = 3.3). Considering that even low concentrations of HNO_2_ suppress nitrification (Anthonisen et al., 1976) the concentration of NO_2_^–^ in the bioreactor was controlled to be as low as possible to avoid the accumulation of HNO_2_. The concentration of NaNO_2_ in the supplied medium was increased in a stepwise way but controlled carefully to completely oxidize all NO_2_^–^ in the bioreactor. Therefore, NO_2_^–^ was completely oxidized to NO_3_^–^ and the NO_3_^–^ concentration in the bioreactor increased as the influent concentration of NO_2_^–^ increased during days 28–303 (Figure 1B). On day 304, the pH was reduced from 6.0 to 5.5. Therefore, the concentration of NO_2_^–^ in the bioreactor rapidly increased to 2.9 mM on day 308. The decrease in nitrite oxidation activity could be due to the increase in HNO_2_ with the change in pH. To avoid the accumulation of HNO_2_ in the bioreactor, we step-wisely reduced the NaNO_2_ concentration in the supplied medium from 3.93 mM to 0.36 mM during days 310–387. Just after decreasing the inflow NO_2_^–^ concentration from 3.93 mM to 1.79 mM, NO_3_^–^ concentration in the bioreactor partially recovered. Furthermore, as the NO_2_^–^ concentration in the inflow medium decreased, the NO_2_^–^ concentration in the bioreactor gradually decreased to reach 0 mM. These results suggest that the nitrite-oxidation activity in the NaNO_2_-fed bioreactor was recovered by adjusting the NO_2_^–^ concentration in the supplied medium, although the oxidation was temporarily inhibited by a decrease in pH. Therefore, we presumed that the sharp decrease in the nitrite oxidation rate could be caused not only by the change in pH, but also by the increase in HNO_2_ concentration.

To investigate the relationship between the nitrification activity and the concentration of NH_3_ or HNO_2_, the theoretical concentration of NH_3_ in the NH_4_Cl-fed bioreactor and HNO_2_ concentration in the NaNO_2_-fed bioreactor were calculated according to the method of a previous study (Anthonisen et al., 1976). In the NH_4_Cl-fed bioreactor controlled at pH 6.0 until day 373, the NH_3_ concentration in the bioreactor increased as the concentration of NH_4_Cl in the supplied medium increased (Figure 1C). When the medium containing 39 mM NH_4_Cl was supplied during days 165–205, the average NH_3_ concentration was 6.6 ± 1.2 μM. Afterward, the NH_4_Cl concentration in the supplied medium increased to 50 mM on day 206 and the average NH_3_ concentration in the bioreactor increased to 14.9 ± 3.2 μM during days 206–373. On day 374, the pH was decreased from 6.0 to 5.5; concurrently the NH_3_ concentration also decreased and kept constant at 7.4 ± 2.0 μM until day 791. Based on this result, the decrease in the nitrification rate after pH change is considered to be partly caused by the decreasing concentration of free ammonia, the substrate for nitrification.

Furthermore, in the NaNO_2_-fed bioreactor controlled at pH 6.0, HNO_2_ temporarily increased up to 2.0 μM on day 142 (Figure 1D). This was due to the sudden increase in the influent NO_2_^–^ concentration. However, at this point, the nitrification rate increased rapidly and the NO_2_^–^ concentration in the bioreactor decreased immediately by day 161. From this result, the nitrifier community in the bioreactor would be still active under the conditions of 1.1 μM HNO_2_ on day 161. In contrast, the nitrification activity decreased immediately after the pH was reduced from 6.0 to 5.5 on day 304. Simultaneously, the HNO_2_ concentration increased to 6.8 μM on day 308 and remained in the bioreactor until day 336. During this period, nitrification in the bioreactor did not recover unless the influent NO_2_^–^ concentration was reduced. Although 6.8 μM of HNO_2_ inhibited nitrification in the bioreactor, the nitrifier community would be still active under pH 5.5. Consequently, it is considered that HNO_2_, rather than low pH, suppressed the nitrification activity.

### Morphology of Nitrifiers

The non-woven fabrics in both bioreactors became brown colored with the biomass (Supplementary Figure S1). The cell suspensions collected from the bioreactors formed flocs visible to naked eyes. The morphological feature of these flocs was similar to an enrichment culture of *Nitrospira* in a previous report (Spieck et al., 2006).

**FIGURE 2:**
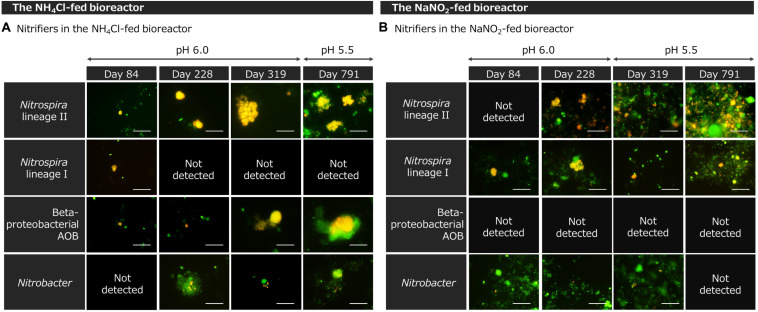
Fluorescence in situ hybridization (FISH) images of enrichment samples from **(A)** the NH_4_Cl-fed bioreactor and **(B)** NaNO_2_-fed bioreactor. All bacteria were stained with SYTOX Green (green). Only targeted cells were labeled with FISH probes (red) and two colors were merged into yellow. The scale bars represent 10 μm.

In the NH_4_Cl-fed bioreactor, *Nitrospira* lineage II and betaproteobacterial AOB grew remarkably (Figure 2A). On day 84, several cells of *Nitrospira* lineage II aggregated and formed small colonies. Such small colonies gathered to form larger dense aggregates on day 228 and 319. Until day 319, those aggregates were mainly composed of *Nitrospira* lineage II cells. However, on day 791, *Nitrospira* lineage II were co-aggregated with colonies of other bacteria. This morphological change in *Nitrospira* lineage II might be caused by the pH decrease. The co-aggregates containing *Nitrospira* lineage II resembled the structures observed in the culture incubating “*Ca.* Nitrospira nitrosa” and “*Ca.* Nitrospira nitrificans” (van Kessel et al., 2015) rather than the small microcolonies of *Nitrospira inopinata* (Daims et al., 2015). In contrast, betaproteobacterial AOB grew throughout the incubation. As in *Nitrospira* lineage II, aggregates mainly composed of AOB cells were observed on day 319 at pH 6.0. However, AOB adhered to other bacteria on day 791 at pH 5.5. Such a co-aggregation of betaproteobacterial AOB with other bacteria was induced by pH reduction in a previous study (De Boer et al., 1991). Unlike the significant growth of *Nitrospira* lineage II and AOB, *Nitrospira* lineage I and *Nitrobacter* were hardly observed throughout the incubation.

Bacteria in the NaNO_2_-fed bioreactor formed strongly aggregated structures with widths of several tens of micrometers (Figure 2B). On day 228, after the stepwise increase in the influent NaNO_2_ concentration, dozens of *Nitrospira* lineage II cells formed colonies. On day 319, just after changing the pH from 6.0 to 5.5, *Nitrospira* lineage II decreased and its colonies became smaller. However, on day 791, after the NaNO_2_ concentration in the bioreactor was kept low, *Nitrospira* lineage II grew again. At pH 5.5, *Nitrospira* lineage II aggregated with other bacteria and formed a complex structure. This aggregation is considered to be an adaptation to lower pH or higher concentrations of HNO_2_. *Nitrospira* was reported to increase the production of extracellular polymeric substances to form aggregates under high nitrite concentrations (Nowka et al., 2015). The structure of *Nitrospira* lineage I aggregates was similar to that of lineage II. Furthermore, betaproteobacterial AOB was under the detection limit of microscopic observation throughout the incubation. In addition, only a small number of *Nitrobacter* cells were observed as a part of the aggregates.

### 16S rRNA Gene-Based Bacterial Community

**FIGURE 3:**
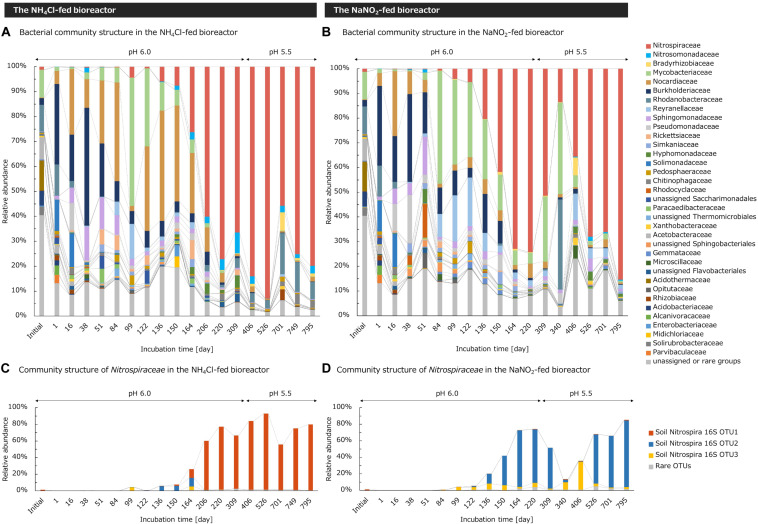
Taxonomic assignments of 16S rRNA gene sequences of enrichment cultures in **(A,C)** the NH_4_Cl-fed bioreactor and **(B,D)** NaNO_2_-fed bioreactor. **(A,B)** Relative abundances of taxonomic groups were analyzed at the family classification level. Unassigned taxon and phylogenetic groups occupying <3% of the total bacteria in all samples were integrated to “unassigned or rare groups.” **(C,D)** Relative abundances of taxonomic groups in the family *Nitrospiraceae* were analyzed at the OTU level. OTUs occupying <2% of the total bacteria in all samples were integrated into “rare OTUs.”

Based on the V7–V8 region of the 16S rRNA gene, the bacterial community structure in the bioreactors was analyzed at the family level. In the initial soil sample, the family *Nitrospiraceae* including comammox accounted for 1.2% of the total bacteria, which was the largest among nitrifying bacteria (Figure 3). In contrast, the family *Nitrosomonadaceae* including betaproteobacterial AOB and the family *Bradyrhizobiaceae* including the genus *Nitrobacter* occupied <0.1% of the total bacteria in the initial soil sample.

In the NH_4_Cl-fed bioreactor, the nitrification activity increased during days 165–205 when the medium containing 39 mM NH_4_Cl was supplied. At the same time, the relative abundance of family *Nitrospiraceae* also increased and reached 60.1% by day 206 (Figure 3A). After that, the abundance of *Nitrospiraceae* fluctuated within 55.8–92.9%, but consistently accounted for more than half of the whole bacterial population, regardless of the pH change. Although another nitrifier oxidizing ammonia, *Nitrosomonadaceae* also increased in number, the abundance remained <8.4% throughout the incubation. The relative abundance of *Bradyrhizobiaceae* including *Nitrobacter* also remained <7.3%. Considering that a small number of AOB existed and a small number of NO_2_^–^ were produced in the bioreactor, some bacteria of the family *Nitrospiraceae* would function as *Nitrospira* oxidizing only NO_2_^–^ and not NH_3_. However, based on the great difference between the proportion of *Nitrosomonadaceae* and that of *Nitrospiraceae*, most of the *Nitrospiraceae* in the bioreactor was assumed to function as comammox *Nitrospira* oxidizing NH_3_ to NO_3_^–^. Since the family *Nitrospiraceae* became dominant in the bioreactor and maintained a stable proportion, the culture condition in this study was considered to be suitable for cultivating *Nitrospiraceae* from an acidic soil. In addition, an isolate of acidophilic AOA from acidic soils was reported to be deactivated at pH 6.0 (Lehtovirta-Morley et al., 2014). Thus, the initial culture condition at pH 6.0 might eliminate AOA in the soils. However, we did not try to detect AOA in the enrichment sample with molecular biological techniques such as quantitative PCR. Therefore, note that the possibility of ammonia oxidation by AOA cannot be denied. Moreover, even though *Nitrospiraceae* was enriched with inorganic medium, some heterotrophic bacteria such as *Rhodanobacteraceae* remained in the bioreactor. *Rhodanobacteraceae* is known as a heterotrophic denitrifying bacterium (Kostka et al., 2012). Those bacteria might consume NH_3_ as nitrogen source or reduce NO_3_^–^ by denitrification to contribute to the inconsistency of stoichiometric ratio of ammonia consumption and nitrate generation (Figure 1A).

Likewise, in the NaNO_2_-fed bioreactor controlled at pH 6.0, the relative abundance of *Nitrospiraceae* increased as the influent NO_2_^–^ concentration gradually increased up to 3.93 mM during days 28–150. After that, the pH in the bioreactor and influent NO_2_^–^ concentration kept a constant level until day 303. In this period, the relative abundance of family *Nitrospiraceae* increased to 72.9% on day 164 and remained an almost constant abundance until day 220 (Figure 3B). However, the nitrification activity of the bioreactor decreased after the pH was decreased from 6.0 to 5.5 on day 304. Concurrently, the proportion of *Nitrospiraceae* decreased from 51.4% on day 309 to 13.5% on day 340. In contrast, the abundance of the family *Bradyrhizobiaceae*, including *Nitrobacter*, increased from 0.32% on day 309 to 7.20% on day 406 following the pH change. This different response between *Nitrospira* and *Nitrobacter* to pH change is considered to be due to the relatively higher resistance of *Nitrobacter* to HNO_2_ (Blackburne et al., 2007). After the decrease in HNO_2_ concentration in the bioreactor, the nitrification was reactivated and the relative abundance of *Nitrospiraceae* recovered to 35.6% on day 406. During days 526–795, the bioreactor operated under a stable environment, and the abundance of *Nitrospiraceae* reached to the range of 66.1%–85.4%. In the environment with a low concentration of HNO_2_, however, the relative abundance of *Bradyrhizobiaceae* was always <1%, much lower than that of *Nitrospiraceae*. From this viewpoint, *Nitrospira* was assumed to be the main contributor to NO_2_^–^ oxidation in the bioreactor at pH 5.5, except for under high concentrations of HNO_2_. In addition, despite the absence of ammonium in the inflow medium supplied to the bioreactor, the family *Nitrosomonadaceae* consisting of AOB grew and comprised 1.82% of the bacterial community on day 526. Although AOB needs NH_3_ as a substrate to grow, most of them are known to produce NH_3_ by degrading urea (Koper et al., 2004; Norton et al., 2008). Therefore, AOB in the bioreactor might degrade the small amount of urea provided by bacteria coexisting in the bioreactor, which was a limited proportion of the biomass.

Furthermore, community structures of the families *Nitrospiraceae*, *Nitrosomonadaceae*, and *Bradyrhizobiaceae* were analyzed at the level of OTUs. *Nitrospiraceae* was mainly composed of three OTUs. Before the cultivation, the most abundant of the three OTUs was soil Nitrospira 16S OTU1, which accounted for 1.0% of the whole bacterial community in the initial soil sample (Figure 3C). Considering that the relative proportion of *Nitrospiraceae* was 1.19% and the total abundance of *Nitrospiraceae*, *Nitrosomonadaceae*, and *Bradyrhizobiaceae* was only 1.25%, soil Nitrospira 16S OTU1 was regarded as the dominant bacteria in the community of nitrifiers in the initial soil sample. In the NH_4_Cl-fed bioreactor, soil Nitrospira 16S OTU1, OTU2, and OTU3 coexisted until day 164, however, soil Nitrospira 16S OTU1 was enriched and accounted for more than half of the bacterial community during days 206–795 (Figure 3C). Based on this result, the continuous-feeding bioreactor supplied with an inorganic medium containing excessive NH_4_Cl at pH 5.5–6.0 seemed to be suitable for enrichment of the major group of *Nitrospira* in acidic soil. On the other hand, the other two OTUs were enriched in the NaNO_2_-fed bioreactor. Soil Nitrospira 16S OTU2, accounting for only 0.02 % in the initial soil, became dominant in the bioreactor on day 164 (Figure 3D). In contrast, the relative abundance of soil Nitrospira 16S OTU3 was lower than 7% throughout the incubation except under high HNO_2_ concentrations. After the pH change and the accumulation of HNO_2_ in the bioreactor, soil Nitrospira 16S OTU3 increased its proportion to 8.7% and 33.8% on days 340 and 406, respectively. Afterward, the soil Nitrospira 16S OTU3 decreased and soil Nitrospira 16S OTU2 became dominant in the bioreactor again. From this result, the factor deciding the niche differentiation between soil Nitrospira 16S OTU2 and OTU3 would be the tolerance to HNO_2_, rather than the pH.

The composition of the family *Nitrosomonadaceae* was also analyzed at the OTU level. The cultivated *Nitrosomonadaceae* was mainly composed of two OTUs. In the NH_4_Cl-fed bioreactor, soil Nitrosospira 16S OTU1 classified into the genus *Nitrosospira* was enriched (Supplementary Figure S2A). This OTU accounted for 0.011% of the total bacterial community in the initial soil and was the dominant OTU of the family *Nitrosomonadaceae* accounting for 0.032%. In the NaNO_2_-fed bioreactor, soil Nitrosospira 16S OTU2 was enriched, but this group was not detected in the initial soil sample (Supplementary Figure S2B). In contrast to *Nitrosomonadaceae*, the communities of family *Bradyrhizobiaceae* in the two bioreactors were composed of a common OTU assigned to the genus *Nitrobacter* (Supplementary Figure S3). This OTU, named soil Nitrobacter 16S OTU1, was the major OTU in *Bradyrhizobiaceae* throughout the cultivation and accounted for 0.032% of the total bacterial community in the initial soil.

### 16S rRNA Gene-Based Phylogenetic Analyses

**FIGURE 4:**
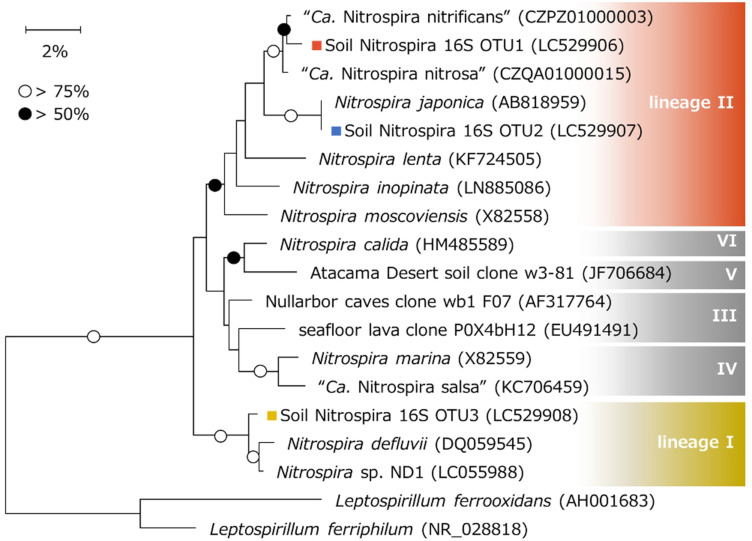
Phylogenetic tree based on the 16S rRNA gene sequences of *Nitrospira*. Phylogenetic analysis was performed using the neighbor-joining method with 1000 bootstraps. Bootstrap values of >75% or >50% are indicated as open or closed circles, respectively. Sequences of *Leptospirillum* were used to root the tree. Colored squares show the OTUs obtained in this study.

A phylogenetic tree of *Nitrospira* based on the 16S rRNA gene sequence was constructed referring to OTUs retrieved from amplicon sequencing and sequences registered in the NCBI nucleotide database. Therefore, soil Nitrospira 16S OTU1 and OTU2 were classified into *Nitrospira* lineage II (Figure 4). Soil Nitrospira 16S OTU1 was grouped within the cluster including “*Candidatus* Nitrospira nitrificans” (van Kessel et al., 2015), an enriched strain of comammox *Nitrospira*, while soil Nitrospira 16S OTU2 was closely related to *Nitrospira japonica*, an isolated strain of nitrite-oxidizing *Nitrospira* (Ushiki et al., 2013). On the other hand, soil Nitrospira 16S OTU3 was classified into lineage I and was closely related to *Nitrospira* sp. ND1 (Fujitani et al., 2013).

In the NaNO_2_-fed bioreactor, soil Nitrospira 16S OTU2 classified into lineage II and soil Nitrospira 16S OTU3 classified into lineage I competed with one another during the continuous-feeding incubation (Figure 3D). Soil Nitrospira 16S OTU2 did not decrease its relative abundance with exposure to 2.03 μM HNO_2_ during days 142–152, but decreased just after being exposed to 6.8 μM HNO_2_ on day 308 (Figures 1D, 3D); meanwhile, the relative abundance of soil Nitrospira 16S OTU3 increased. From these results, *Nitrospira* lineage II in the bioreactor was presumed to be advantageous under low concentrations of NO_2_^–^ regardless of pH change; however, it was more sensitive to HNO_2_ than lineage I. By contrast, lineage I was estimated to be favorable under relatively high concentrations of HNO_2_ and tolerated 6.8 μM HNO_2_ (Table 1). Moreover, a similar niche differentiation between the two lineages was reported in previous studies cultivating *Nitrospira* from activated sludge under different NO_2_^–^ concentrations (Maixner et al., 2006; Fujitani et al., 2013). Furthermore, the concentration of diluted oxygen (Park and Noguera, 2008) and usability of organic substrates (Gruber-Dorninger et al., 2015) were reported as the niche differentiation factors between the two lineages. However, all of these theories are based on experiments using samples from wastewater treatment systems. Therefore, the enrichment sample obtained in this study would provide important information to reveal the factors influencing the niche differentiation between the two lineages in acidic conditions and soil environments.

**TABLE 1:** Features of the OTUs obtained from amplicon sequencing of the 16S rRNA gene and comammox *amoA* gene.

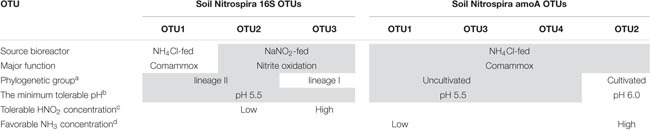

**FIGURE 5:**
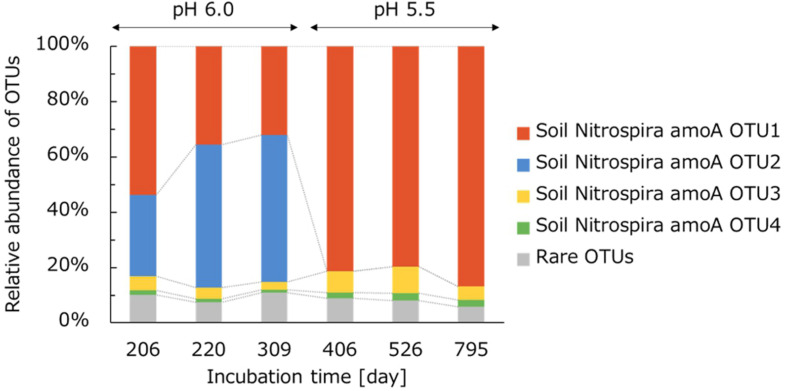
Relative abundance of taxonomic groups in comammox *Nitrospira* in the NH_4_Cl-fed bioreactor. Taxonomic groups were analyzed based on comammox amoA gene sequences at the OTU classification level. OTUs occupying <2% of the total comammox community in all samples were integrated into “rare OTUs.”

**FIGURE 6:**
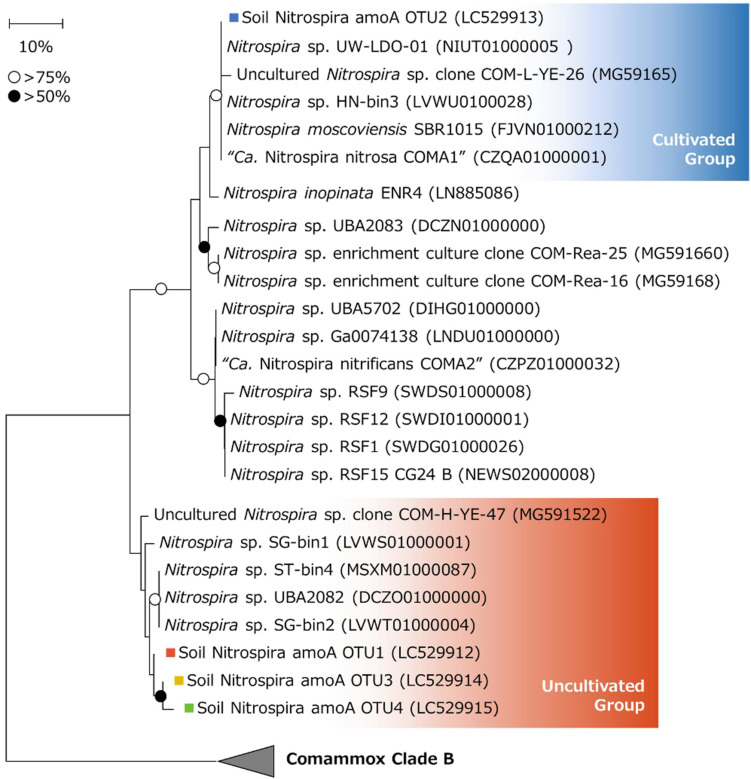
Phylogenetic tree of comammox clade A based on AmoA amino acid sequences translated from nucleotide sequences. Phylogenetic analysis was performed using the neighbor-joining method with 1000 bootstraps. Bootstrap values of >75% or >50% are indicated as open or closed circles, respectively. Colored squares show the OTUs obtained in this study. Comammox clade B sequences (NEWT02000035, NEWR02000002, OEKW00000000, and OELE00000000) were used as outgroups.

*The gray cells represent common features of several OTUs. Empty cells represent no data. ^*a*^OTUs were classified based on phylogenetic analyses of the 16S rRNA gene sequences (Figure 4) and AmoA amino acid sequences (Figure 6). ^*b*^All soil Nitrospira 16S OTUs were detected throughout the incubation regardless of the pH decrease from 6.0 to 5.5 (Figures 3C,D). Likewise, soil Nitrospira amoA OTU1, OTU3, and OTU4 were detected at pH 5.5 (Figure 5). On the other hand, soil Nitrospira amoA OTU2 was detected at only pH 6.0 (Figure 5). ^*c*^Soil Nitrospira 16S OTU2 decreased its relative abundance with exposure to 6.8 μM HNO_2_ on day 308 (Figures 1D, 3D); meanwhile, the relative abundance of soil Nitrospira 16S OTU3 increased. From these results, soil Nitrospira 16S OTU3 would be tolerable to relatively high concentration of HNO_2_. ^*d*^The concentration of NH3 in the NH_4_Cl-fed bioreactor was 6.6 ± 1.2 μM during days 165–205 (Figure 1C). Just after this period, on day 206, soil Nitrospira amoA OTU1 dominated among the comammox community (Figure 5). Afterwards, the NH3 concentration increased to 14.9 ± 3.2 μM during days 206–373 (Figure 1C). In this period, soil Nitrospira amoA OTU2 became dominant instead (Figure 5). Thus, soil Nitrospira amoA OTU2 could be adapted to relatively high NH3 concentration.*

While *Nitrospira* lineage I contains only nitrite-oxidizing *Nitrospira*, lineage II also includes comammox. Soil *Nitrospira* 16S OTU1 was enriched in the NH_4_Cl-fed bioreactor but was rarely detected in the NaNO_2_-fed bioreactor (Figure 3). Furthermore, according to metagenomic studies, comammox *Nitrospira* are known to lack assimilatory nitrite reductase or cyanate hydratase genes for generating ammonia, while nitrite-oxidizing *Nitrospira* do contain these genes and are able to grow in inorganic media containing only NO_2_^–^ as a nitrogen source (Palomo et al., 2018). In contrast, most of the comammox genomes harbor urease genes to produce ammonia through urea degradation (Palomo et al., 2018), which might enable the growth of comammox depending on urea produced by other bacteria. However, urea-dependent ammonia oxidation is known to take longer than ammonia oxidation under NH_4_Cl supplementation (Pommerening-Roser and Koops, 2005). Based on these facts, soil Nitrospira 16S OTU1 could be composed of comammox *Nitrospira.* In contrast, soil Nitrospira 16S OTU2 enriched in the NaNO_2_-fed bioreactor was thought to mainly include nitrite-oxidizing *Nitrospira*. However, note that the phylogenetic position does not directly support the function of complete ammonia oxidation (Pjevac et al., 2017).

To investigate the phylogenetical positions of OTUs classified into the genera *Nitrosospira* and *Nitrobacter*, the related sequences were searched using NCBI BLAST. Soil Nitrosospira 16S OTU1 was 100% identical to the 16S rRNA gene sequence of *Nitrosospira lacus* strain APG3. This strain was reported to grow under a wide range of pH values (5–9) (Urakawa et al., 2015). Although APG3 was isolated from a freshwater lake (Garcia et al., 2013), closely related *Nitrosospira* was also detected in a soil (Zhang et al., 2015). In contrast, soil Nitrosospira 16S OTU2 cultivated in the NaNO_2_-fed bioreactor was closely related to *Nitrosospira* sp. EnI299 enriched from fertilized soil (Tourna et al., 2010). Moreover, soil Nitrobacter 16S OTU1 was 100% identical to the 16S rRNA gene sequence of *Nitrobacter* sp. Io acid and *Nitrobacter vulgaris*. *Nitrobacter* sp. Io acid was isolated from an acidic forest soil and showed nitrite-oxidizing activity at pH 3.5–7.0 (Hankinson and Schmidt, 1988). *Nitrobacter vulgaris* DSM10236 was cultivated in media with neutral or mildly alkaline pH, such as 7.4 or 8.6 (Reshetilov et al., 2011). For a different strain of the same species, *Nitrobacter vulgaris* strain mesi survived in a fairly wide range of pH values (6.0–9.2) (Laanbroek and Schotman, 1991). Therefore, the adaptable pH range for *Nitrobacter* depends on each strain and it is difficult to distinguish the range based solely on the 16S rRNA gene sequence at this point.

### *amoA* Gene-Based Bacterial Community of Comammox *Nitrospira*

As described above, *Nitrospira* lineage II includes both comammox and nitrite-oxidizing *Nitrospira*; it is difficult to distinguish these two groups based on 16S rRNA gene sequences (Pjevac et al., 2017). To analyze the phylogenetic features of comammox *Nitrospira* specifically, an amplicon sequencing targeted at the comammox *amoA* gene was performed. However, the comammox *amoA* gene in the extracted DNA samples was not amplified before day 206 (data not shown). The impossibility of amplification could be due to the low abundance of comammox *Nitrospira* and low PCR efficiency of barcode-attached primers. The relative abundance of each OTU was calculated as a proportion to the total reads of comammox *amoA* sequences. The comammox *Nitrospira* community in the NH_4_Cl-fed bioreactor was mainly composed of four OTUs. Other OTUs accounting for less than 2% of all samples were integrated into “rare OTUs.” Especially, two OTUs, named soil Nitrospira amoA OTU1 and soil Nitrospira amoA OTU2 were dominant (Figure 5). During days 206–309, the pH in the bioreactor was controlled at 6.0 and the concentration of NH_4_Cl in the influx medium was maintained at 50 mM. In this period, the relative abundance of soil Nitrospira amoA OTU2 slowly increased, while that of soil Nitrospira amoA OTU1 decreased (Figure 5). After the pH change from 6.0 to 5.5, however, soil Nitrospira amoA OTU2 was not detected and soil Nitrospira amoA OTU1 became dominant instead.

The dynamics of the relative abundances of comammox OTUs caused by the pH change indicated that the soil Nitrospira amoA OTU2 activity was suppressed or completely inhibited at pH 5.5. The acidic condition stresses microorganisms by affecting pH homeostasis or decreasing HCO_3_^–^ as a carbon source (Lehtovirta-Morley et al., 2016; Herbold et al., 2017). Thus, the pH decrease might suppress the growth of Nitrospira amoA OTU2. The concentration of NH_3_ in the bioreactor controlled at pH 5.5 during days 374–791 corresponded to the value at pH 6.0 during days 165–205 (Figure 1C). Just after this period, on day 206, soil Nitrospira amoA OTU2 accounted for a high proportion of the comammox community. Based on this trend, the decrease in NH_3_ concentration caused by the pH change on day 374 would not be critical to the nitrification activity of soil Nitrospira amoA OTU2. Indeed, soil Nitrospira amoA OTU2 was unable to grow under pH 5.5 like typical ammonia oxidizers (De Boer and Kowalchuk, 2001). Instead, soil Nitrospira amoA OTU1 became dominant under relatively acidic conditions at pH 5.5.

Moreover, unlike the dynamic change in the comammox *amoA* gene-based community structure, 16S rRNA gene-based analysis of the *Nitrospira* community showed no significant change before and after the pH decrease (Figure 3C). The gap between these two analyses was considered to be caused by the difference in the similarities of nucleotide sequences. Partial 16S rRNA gene sequences of nitrifiers have high similarities, and thus, it is difficult to distinguish between closely related bacteria. In contrast, functional marker genes are considered to be suitable for analyzing the diversity in detail and characterizing the phenotypic features (Norton et al., 2002). Especially, the *amoA* gene has been used as a suitable functional marker gene to analyze the phylogeny of comammox *Nitrospira* because it enables comammox and nitrite-oxidizing *Nitrospira* to be differentiated (Yu et al., 2018). In this study, analysis based on the *amoA* gene sequence detected several groups of comammox *Nitrospira*. Generally, genomes of comammox *Nitrospira* harbor one single copy of the *amoA* gene (Palomo et al., 2018). Therefore, several types of comammox were presumed to exist in the bioreactor. Even though *amoA* gene sequences detected in this experiment were classified into different OTUs, amplicon sequencing of the 16S rRNA gene found only one OTU in the NH_4_Cl-fed bioreactor. This could be because the phylogenetically distinct types of comammox at the *amoA* gene level shared a high proportion of 16S rRNA gene sequences, and they were grouped into an identical OTU. This impossibility of linking each *amoA* OTU with one specific 16S rRNA gene sequence could be solved by using a single-cell isolation system combined with the *de novo* assembly of single-cell genomes (Chitsaz et al., 2011).

### AmoA-Based Phylogenetic Analysis of Comammox *Nitrospira*

The nucleotide sequences of comammox *amoA* OTUs were translated into amino acid sequences. A phylogenetic tree was constructed by referring to these translated sequences and those registered in the NCBI database. Comammox AmoA are known to be divided into two clades, and the primer pair used in this study was able to amplify the *amoA* genes of both clade A and B (Fowler et al., 2018). However, all of the detected OTUs were classified into clade A. Looking in further detail, soil Nitrospira amoA OTU2 and other OTUs were classified into different branches of clade A (Figure 6). The phylogenetical gap between these two groups corresponded to the difference in low pH adaptation; soil Nitrospira amoA OTU2 was deactivated at pH 5.5, while the other three OTUs survived (Table 1). Moreover, soil Nitrospira amoA OTU2 was grouped into the same branch with a cultivated comammox *Nitrospira* reported in previous studies (Daims et al., 2015; van Kessel et al., 2015; Camejo et al., 2017; Yu et al., 2018). On the other hand, soil Nitrospira amoA OTU1, OTU3, and OTU4 were classified into another branch with uncultured *Nitrospira*. Genes encoding AmoA of those *Nitrospira* were detected in drinking water (Wang et al., 2017), marine sediments (Parks et al., 2017) and river sediments (Yu et al., 2018). In the study cultivating sediments by Yu et al., uncultured *Nitrospira* sp. Clone COM-H-YE-47 was found. Interestingly, however, no identical *amoA* gene sequence was detected in a NH_4_Cl-fed bioreactor incubating the sediment sample (Yu et al., 2018). From these results, it could be inferred that the continuous-feeding bioreactor in this study enabled the enrichment of a group of comammox *Nitrospira* that were fastidious and not cultivated in previous studies. Furthermore, these enriched comammox *Nitrospira* were not only physiologically novel, but also phylogenetically distinct from the strains cultivated in previous researches.

### Effect of the NH_4_Cl Concentration and pH on the Nitrification Activity of the Enrichment Sample

**FIGURE 7:**
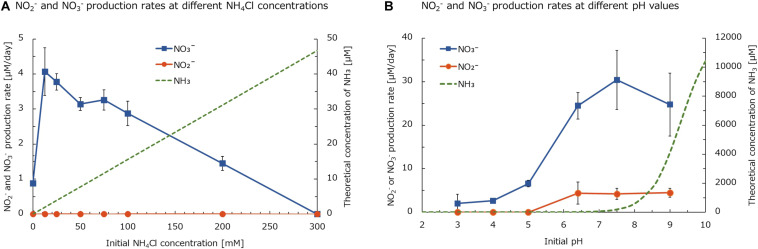
**(A)** NO_2_^–^ and NO_3_^–^ production rates of the enrichment sample from the NH_4_Cl-fed bioreactor at different NH_4_Cl concentrations. The initial pH of media was adjusted to 5.5. **(B)** NO_2_^–^ and NO_3_^–^ production rates of the enrichment sample from the NH_4_Cl-fed bioreactor at different pH values. The medium was supplemented with 12.5 mM of NH_4_Cl. Data show means and error bars show standard deviation of biological triplicates. Green dashed lines show the theoretically calculated concentrations of NH_3_.

To evaluate the nitrification activity of the enrichment sample in the NH_4_Cl-fed bioreactor, the culture sample was collected on day 749 and the activity was tested under different NH_4_Cl concentrations. The initial pH of the medium was adjusted to pH 5.5, the same value as the medium in the bioreactor. Furthermore, the NH_4_Cl concentration in the medium was adjusted at 0, 12.5, 25, 50, 75, 100, 200, or 300 mM. After three days of incubation, NO_3_^–^ was accumulated in the samples, but NO_2_^–^ was rarely detected (Figure 7A). This result indicated that all the oxidized ammonia was converted to nitrate. Even in the sample not supplemented with NH_4_Cl, NO_3_^–^ was slightly generated. This is likely because a small amount of ammonium was produced by the degradation of organic nitrogen such as cyanate or urea. Considering the culture sample containing heterotrophic bacteria other than chemolithoautotrophic nitrifiers, the amount of organic nitrogen generated from the biomass could not be negligible.

The NO_3_^–^ production rate was the highest at a NH_4_Cl concentration of 12.5 mM (Figure 7A). The theoretical concentration of free ammonia (NH_3_) under this condition was calculated to be 1.94 μM. This value is smaller than 33.3 μM, the half saturation constant (*K*_m_) of NH_3_ reported for “*Ca.* Nitrosoglobus terrae”, AOB isolated from acidic soil (Hayatsu et al., 2017). On the other hand, AOA isolated from acidic soil, “*Ca.* Nitrosotalea devanaterra”, showed the highest growth rate with 0.18 nM NH_3_ (Lehtovirta-Morley et al., 2011). Therefore, the optimum NH_3_ concentration for comammox *Nitrospira* enriched in this study would be higher than that for AOA but lower than that for AOB cultured from acidic soils. Based on these results, the niche differentiation among terrestrial comammox *Nitrospira*, AOB, and AOA is considered to partly depend on a favorable NH_3_ concentration. This trend corresponds to the difference in affinity for NH_3_, described in a previous study testing the physiological characteristics of *Nitrospira inopinata* (Dimitri et al., 2017). Moreover, the *K*_m_ value of NH_3_ reported for *Nitrospira inopinata* is around 49 to 83nM (Dimitri et al., 2017). Although this value is lower than 1.94 μM, the optimum concentration for the enrichment in this study, it is still larger than that of terrestrial AOA.

Furthermore, the nitrification rate of the enrichment from the NH_4_Cl-fed bioreactor decreased as the initial NH_4_Cl concentration increased (Figure 7A). The enrichment was still active at 200 mM NH_4_Cl. However, the nitrification activity was inhibited by 300 mM NH_4_Cl, which was equivalent to 46.7 μM NH_3_ at pH 5.5. This value almost matches the *K*_m_ of NH_3_ for “*Ca.* Nitrosoglobus terrae”, as described above (Hayatsu et al., 2017). Considering that an acid-adapted AOB could tolerate this amount of NH_3_, the nitrification at pH 5.5 in this experiment was mainly performed by comammox rather than AOB, although the inhibition of comammox *Nitrospira* caused by a high concentration of NH_3_ has not been reported by physiological experiments with pure cultures.

Moreover, to investigate the adaptation to pH change, the nitrification activity of the enrichment sample from the NH_4_Cl-fed bioreactor was tested under a variety of pH values. After 2 days of incubation, the decrease in pH was kept within 1.0 in all samples (data not shown). The enrichment sample oxidized NH_3_ to NO_3_^–^ at a wide range of pH values (3.0–9.0). Within pH 6.4–9.0, NH_3_ was not completely oxidized and NO_2_^–^ was accumulated in the culture (Figure 7B). However, at this pH range, NH_3_ was oxidized relatively faster than at pH 3.0–5.0. Under higher pH conditions, the concentration of NH_3_ increases because of deprotonation. Especially at pH 7.5 and 9.0, the theoretical concentration of NH_3_ was 192 and 4,122 μM, respectively. These values are much higher than the 46.7 μM inhibiting the nitrification activity of the enrichment at pH 5.5 (Figure 7A). Based on these results, the nitrifier oxidizing NH_3_ to NO_3_^–^ at pH 7.5–9.0 would be different from the major nitrifier oxidizing NH_3_ at pH 5.5.

According to the 16S rRNA gene-based analysis, betaproteobacterial AOB enriched in the bioreactor was closely related to *Nitrosospira lacus*. *Nitrosospira lacus* was reported to oxidize NH_3_ under a broad range of pH values (5–9) (Urakawa et al., 2015). Furthermore, the NH_3_ oxidation activity of *Nitrosospira lacus* sharply decreased at pH 6 (Ishii et al., 2017). This trend corresponds to the low NO_3_^–^ production rate at pH 3.0–5.0 in this study (Figure 7B). On the other hand, the slow NO_3_^–^ production at pH 3.0–5.0 would be carried out by comammox *Nitrospira*. The NO_3_^–^ production rate in this pH range was almost equivalent to the rate at pH 5.5 measured in the former experiment (Figure 7A). Therefore, NH_3_ might be mainly oxidized by comammox *Nitrospira* and *Nitrosospira* at pH 3.0–5.0 and 6.4–9.0, respectively. Thus, the gap in the NH_3_ oxidation rate between these two pH ranges might be caused by the difference in the maximum oxidation rate between AOB and comammox *Nitrospira*. According to a previous report, the maximum rate of ammonia oxidation of *Nitrosomonas europaea*, a strain of betaproteobacterial AOB, was several times higher than that of *Nitrospira inopinata* (Dimitri et al., 2017). From these results, the rapid NH_3_ oxidation at pH 6.4–9.0 was considered to be caused by AOB, although the relative abundance of *Nitrosospira* was lower than that of *Nitrospira* in the enrichment sample. Within pH 6.4–9.0, NO_2_^–^ was accumulated in the medium, which also supports two-step oxidation from NH_3_ to NO_3_^–^ via NO_2_^–^ by AOB and NOB. Considering the accumulation of NO_2_^–^ in this experiment, the NOB activity in the enrichment sample might not be sufficient to completely oxidize NO_2_^–^. This could be partly because the abundance of *Nitrobacter* was lower than that of *Nitrosospira* in the enrichment sample. On the other hand, NO_2_^–^ was not detected at pH 3.0–5.0. This result suggests that the complete nitrification occurred at pH 3.0–5.0. However, NO_2_^–^ can be converted to NO_3_^–^ abiotically at pH < 3.3 (Cai et al., 2001). Thus, the complete nitrification at pH 3.0 is still debatable.

In this study, NH_3_ oxidation at pH 3.0–5.0 seemed to be carried out by comammox *Nitrospira*. According to previous studies, ammonia oxidizers cultured from acidic soils, “*Ca.* Nitrosoglobus terrae” and “*Ca.* Nitrosotalea devanaterra”, were unable to oxidize NH_3_ at pH 3.5. From these results, comammox *Nitrospira* enriched in this study would have an equal or higher tolerance to low pH as acid-adapted AOB and AOA. Even though the contribution of each microbe to the nitrification in this experiment was still unclear, this study gives evidence of the ability of diverse nitrifying bacteria communities in soil to adapt to a wide range of pH values.

## Conclusion

In this study, we selectively enriched both comammox and nitrite-oxidizing *Nitrospira* from acidic soils of a tea field fertilized with a large amount of nitrogen. A phylogenetical analysis based on the 16S rRNA gene revealed the niche differentiation between nitrite-oxidizing *Nitrospira* lineage I and lineage II depending on the HNO_2_ concentration rather than pH. Moreover, the amplicon sequencing targeting the *amoA* gene detected OTUs related to uncultivated comammox *Nitrospira*. The enrichment sample of comammox *Nitrospira* showed nitrification activity even at pH 3–4. Therefore, the enriched comammox *Nitrospira* was not only phylogenetically novel, but also might possess the potential to adapt to low pH.

Although *Nitrospira*, as either comammox or NOB, is still difficult to cultivate, physiological studies are necessary to understand their characteristics. This study provides a scientific basis to establish a cultivation method for *Nitrospira* from acidic soils. Furthermore, the enrichment sample enables us to examine the metabolism of *Nitrospira* by comprehensive analyses based on metatranscriptomics or metaproteomics. These approaches could reveal the still unknown acid-adaptation mechanisms of *Nitrospira* and the regulation factors that affect these systems.

## Data Availability Statement

The amplicon sequence data have been deposited in DDBJ Sequence Read Archive with BioProject number PRJDB9437. The OTUs acquired from amplicon sequencing based on the 16S rRNA gene and amoA gene were registered at DDBJ/ENA/GenBank with accession numbers LC529906, LC529907, LC529908, LC529909, LC529910, LC529911, LC529912, LC529913, LC529914, and LC529915.

## Author Contributions

YT, HF, and ST contributed to the conception and design of the study, analyzed the phylogenetical and physiological data, and wrote the manuscript with help from all co-authors. YT cultivated the soil sample and performed all of the experiments using enrichment cultures. YH collected the soil sample. KT, YW, and MH analyzed and identified the chemical characteristics of the soil sample. All authors contributed to the article and approved the submitted version.

## Conflict of Interest

The authors declare that the research was conducted in the absence of any commercial or financial relationships that could be construed as a potential conflict of interest.

## Footnotes

1 https://blast.ncbi.nlm.nih.gov/Blast.cgi

2 https://www.ncbi.nlm.nih.gov/nucleotide/
