# Supplementary material for: Enrichment of Comammox and Nitrite-Oxidizing Nitrospira From Acidic Soils
Source: Front Microbiol. 2020 Jul 22;11:1737. doi: 10.3389/fmicb.2020.01737 (PMC7396549; doi:10.3389/fmicb.2020.01737)
Supplement: Supplementary file 1 [file Data_Sheet_1.PDF]

Supplementary Material

Table S1 | Property of soil used in this study. Standard deviations were calculated from the triplicate of three samples collected from the same point

Soil moisture content (%)	pH	Total C (%)	Total N (%)	Ammonium concentration (μg N g^{-1} of dry soil)	Nitrate concentration (μg N g^{-1} of dry soil)
62.34 ± 0.36	3.67 ± 0.01	28.63 ± 0.60	2.55 ± 0.03	1035.41 ± 36.30	50.76 ± 10.21

Table S2 | The components of medium supplied into the NH_4Cl-fed bioreactor.

Substance	Concentration
NH_4Cl	3.8–2,670 mg L^{-1}
$NaCl$	116 mg L^{-1}
$MgSO_4 \cdot 7H_2O$	40 mg L^{-1}
$CaCl_2 \cdot 2H_2O$	73 g L^{-1}
KCl	38 g L^{-1}
KH_2PO_4	34 g L^{-1}
$FeCl_2 \cdot 6H_2O$	2 mg L^{-1}
EDTA	4.3 mg L^{-1}
$MnCl_2 \cdot 4H_2O$	0.1 mg L^{-1}
$CoCl_2 \cdot 6H_2O$	24 μg L^{-1}
$NiCl_2 \cdot 6H_2O$	24 μg L^{-1}
$CuCl_2 \cdot 2H_2O$	17 μg L^{-1}
$ZnCl_2$	68 μg L^{-1}
$Na_2WO_4 \cdot 2H_2O$	33 μg L^{-1}
Na_2MoO_4	24 μg L^{-1}
H_3BO_3	62 μg L^{-1}

Table S3 | The components of medium supplied into the $NaNO_2$-fed bioreactor.

Substance	Concentration
$NaNO_2$	4.9–271 mg L^{-1}
K_2HPO_4	25 mg L^{-1}
$MgSO_4 \cdot 7H_2O$	41 mg L^{-1}
$CaCl_2 \cdot 2H_2O$	7 mg L^{-1}
$FeSO_4 \cdot 7H_2O$	3 mg L^{-1}
$NaHCO_3$	200 mg L^{-1}
$MnSO_4 \cdot 5H_2O$	54 µg L^{-1}
H_3BO_3	49 µg L^{-1}
$ZnSO_4 \cdot 7H_2O$	43 µg L^{-1}
$Na_2Mo_4O_4$	28 µg L^{-1}
$CuSO_4 \cdot 5H_2O$	25 µg L^{-1}

Table S4 | List of probes used for fluorescence *in situ* hybridization (FISH) in this study

Probe name	Sequence (5'-3')	Target group	Formamide concentration [%]	Reference
S-*-Ntspa-1151-a-A-20	TTC TCC TGG GCA GTC TCT CC	*Nitrospira* lineage II	35	Maixner et al., 2006
S-*-Ntspa-1431-a-A-18	TTG GCT TGG GCG ACT TCA	*Nitrospira* lineage I	35	Maixner et al., 2006
S-G-Nbac-1035-a-A-18*	CCT GTG CTC CAT GCT CCG	*Nitrobacter*	40	Wagner et al., 1996
S-F-bAOB-1224-a-A-20	CGC CAT TGT ATT ACG TGT GA	Betaproteobacterial AOB	35	Mobarry et al., 1996

*A competitor unlabeled probe (CCT GTG CTC CAG GCT CCG) was added to increase the hybridization specificity of the labeled probe.

Table S5 | List of primers used in this study

Primer name	Sequence (5′-3′)	Target group	Usage	Reference
1055f[a]	ATG GCT GTC GTC AGC T	Bacterial 16S rRNA	16S amplicon sequencing	Ferris et al., 1996
1392r[a]	ACG GGC GGT GTG TAC	Bacterial 16S rRNA	16S amplicon sequencing	Lane et al., 1991
Ntsp-amoA 162F[b]	GGA TTT CTG GNT SGA TTG GA	*Nitrospira amoA*	Quantitative PCR/ amoA amplicon sequencing	Fowler et al., 2018
Ntsp-amoA 359R[b]	WAG TTN GAC CAC CAS TAC CA	*Nitrospira amoA*	Quantitative PCR/ amoA amplicon sequencing	Fowler et al., 2018

Thermal cycling protocol:
a. 95°C for 5 min, 30 cycles of 95°C for 30 s, 52°C for 30 s, 72°C for 30 s, followed by 72°C for 4 min.
b. 95°C for 10 min, 40 cycles of 95°C for 45 s, 48 °C for 30 s, 72°C for 45 s, followed by 72°C for 7 min.

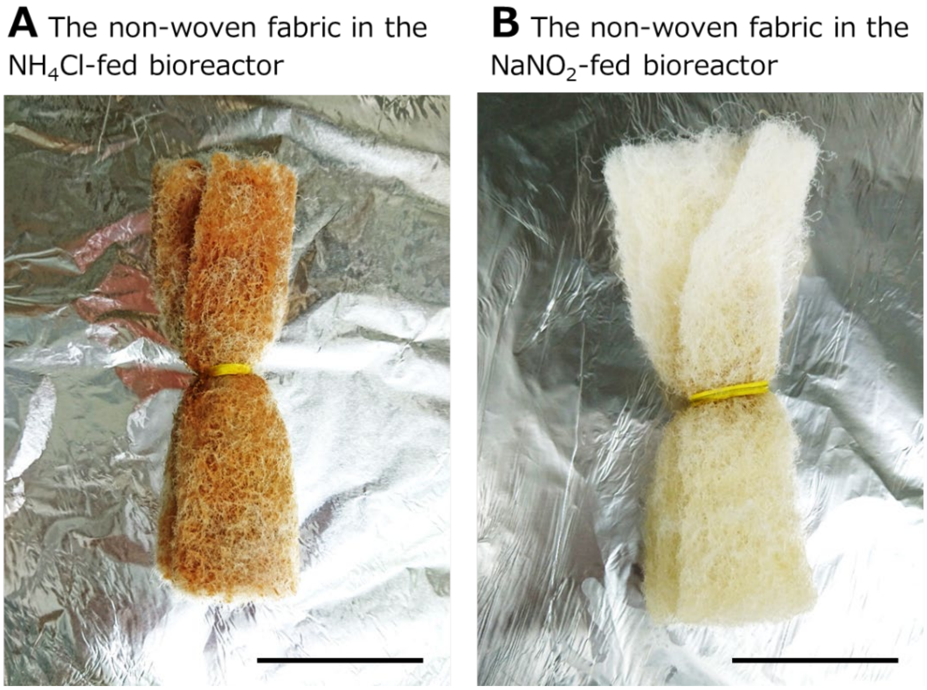

Figure S1. The biomass attached to non-woven fabrics in (A) the NH_4Cl-fed bioreactor and (B) $NaNO_2$-fed bioreactor. The scale bars represent 50 mm.

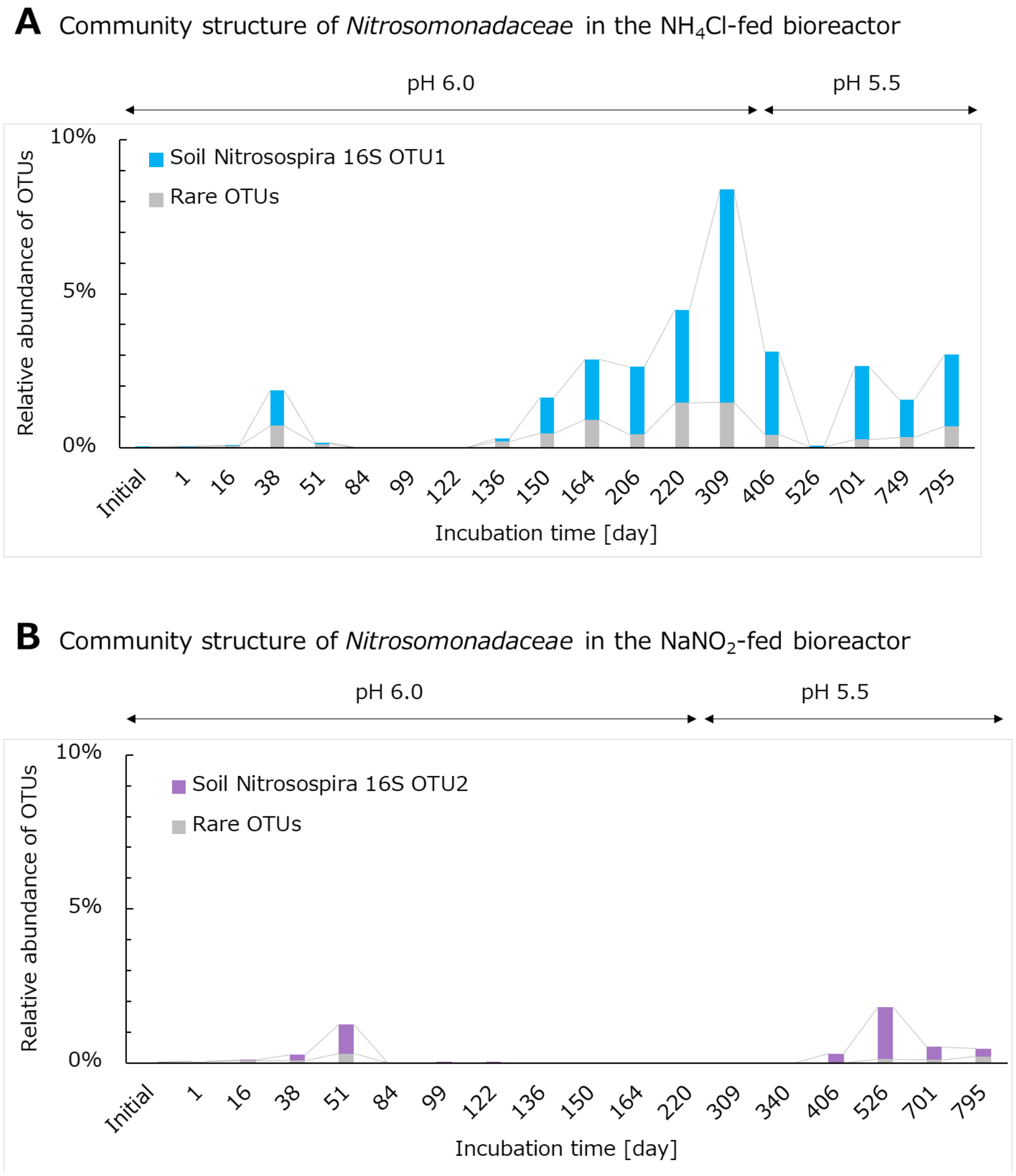

Figure S2. Relative abundance of taxonomic groups classified in the family *Nitrosomonadaceae* in (A) the NH_4Cl-fed bioreactor and (B) $NaNO_2$-fed bioreactor. Taxonomic groups were analyzed based on 16S rRNA gene sequences at the operational taxonomic unit (OTU) classification level. OTUs occupying <1% of the total bacteria in all samples were integrated into “rare OTUs”.

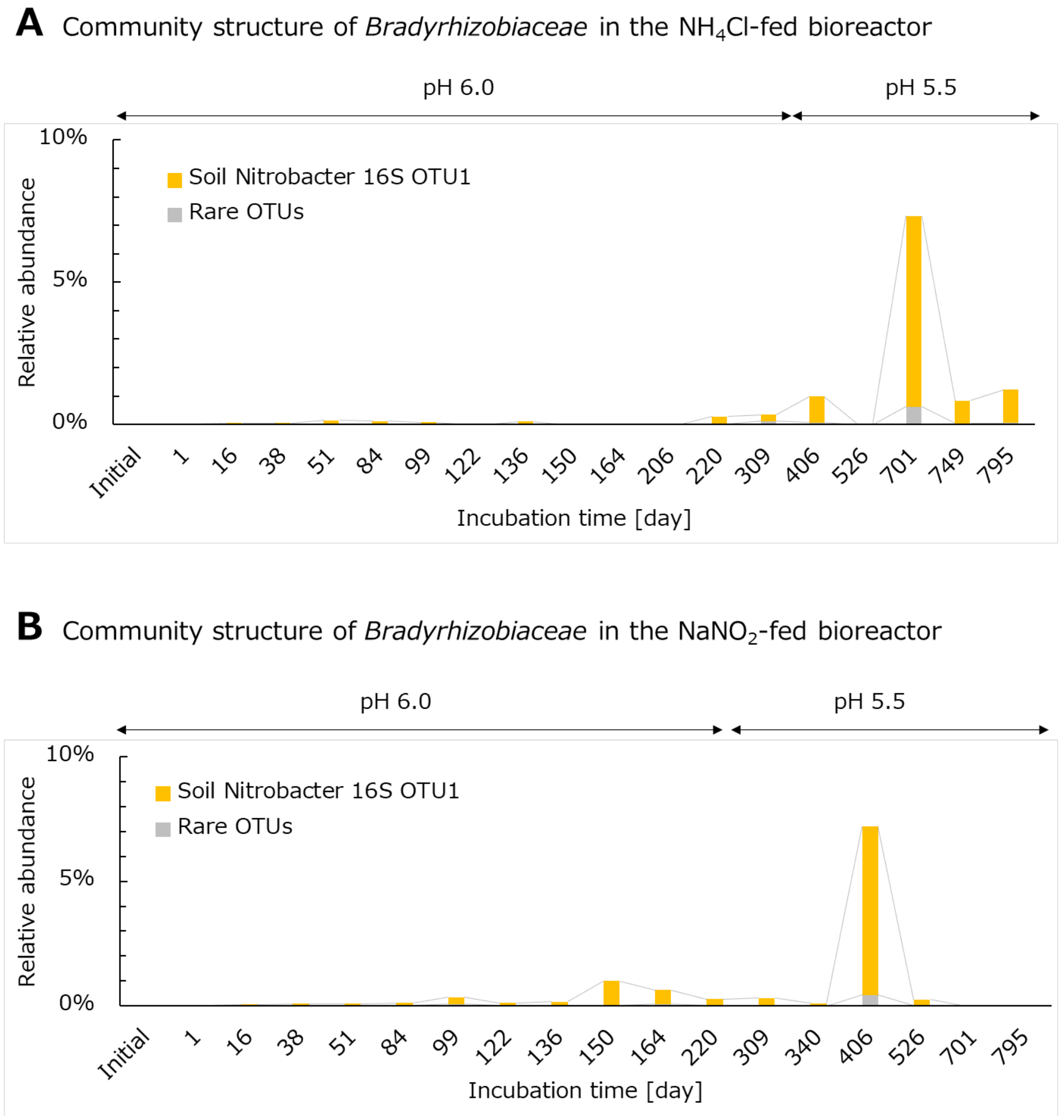

Figure S3. Relative abundance of taxonomic groups classified in the family *Bradyrhizobiaceae* in (A) the NH_4Cl-fed bioreactor and (B) $NaNO_2$-fed bioreactor. Taxonomic groups were analyzed based on 16S rRNA gene sequences at the operational taxonomic unit (OTU) classification level. OTUs occupying <1% of the total bacteria in all samples were integrated into “rare OTUs”.

References

Ferris MJ, Muyzer G, Ward DM (1996). Denaturing gradient gel electrophoresis profiles of 16S rRNA-defined populations inhabiting a hot spring microbial mat community. *Appl Environ Microbiol* 62: 340-346.

Fowler SJ, Palomo A, Dechesne A, Mines PD, Smets BF (2018). Comammox *Nitrospira* are abundant ammonia oxidizers in diverse groundwater-fed rapid sand filter communities. *Environ Microbiol* 20: 1002-1015.

Lane DJ (1991). 16S/23S rRNA sequencing. *Nucleic Acid Techniques in Bacterial Systematics*: 115–75.

Maixner F, Noguera DR, Anneser B, Stoecker K, Wegl G, Wagner M, Daims H (2006). Nitrite concentration influences the population structure of Nitrospira-like bacteria. *Environ Microbiol* 8: 1487-1495.

Mobarry BK, Wagner M, Urbain V, Rittmann BE, Stahl DA (1996). Phylogenetic probes for analyzing abundance and spatial organization of nitrifying bacteria. *Appl Environ Microbiol* 62: 2156-2162.

Wagner M, Rath G, Koops HP, Flood J, Amann R (1996) *In situ* analysis of nitrifying bacteria in sewage treatment plants. *Water Sci & Technol* 34: 237-244
